# Anaemia among adolescent girls in three districts in Ethiopia

**DOI:** 10.1186/s12889-019-6422-0

**Published:** 2019-01-21

**Authors:** Seifu Hagos Gebreyesus, Bilal Shikur Endris, Getahun Teka Beyene, Alinoor Mohamed Farah, Fekadu Elias, Hana Nekatebeb Bekele

**Affiliations:** 1 Department of Reproductive Health and Health Service Management, School of Public Health, College of Health Sciences, Addis Ababa University, Addis Ababa, Ethiopia; 2 World Health Organization (WHO) Ethiopia Country office, Addis Ababa, Ethiopia; 3 Department of Public Health, College of Medicine and Health Sciences, Jigjiga University, Jigjiga, Ethiopia; 4 School of Public Health, College of Health Sciences, Sodo University, Sodo, Ethiopia

**Keywords:** Anaemia, Adolescent girls, Ethiopia, Iron folic acid, Food insecurity

## Abstract

**Background:**

Adolescence is characterized by rapid growth and development with a significantly increased need for macro and micronutrients. However, there is little empirical evidence on the burden of anaemia among adolescent girls in developing countries such as Ethiopia. This study aims to address this gap by evaluating the magnitude of anaemia with an aim to guide design of intervention modalities to address anaemia among adolescent girls.

**Methods:**

The study employed a community based cross sectional design. The study was conducted on weekends to capture both in school and out of school adolescent girls. Data was collected from a total 1323 adolescent girls. From each district, we randomly selected villages and ensured that the sampled households had a range geographical spread (lowlands, highlands) within the larger category of rural and urban. We performed anaemia testing using HemoCue B-Haemoglobin analyser. We applied a complex survey data analysis method to estimate the level of anaemia. The hemoglobin level was adjusted for altitude and smoking status. We ran a logistic regression model to evaluate predictors of anaemia.

**Results:**

The overall anaemia prevalence ranged from 24 to 38%, with an average rate of 29%. Less than half of the girls heard the term anaemia, and about one third knew the relationship between anaemia and the intake of iron rich foods. The risk of anaemia is higher among adolescent girls in their early adolescence period (10–14 years) (Adjusted Odds Ratio (AOR); 1.98; 95% CI; 1.03, 3.82] and among adolescent girls who lived in moderately food insecure households (AOR 1.48; 95% CI; 1.05–2.09). However, knowing the term “*anaemia”* was found to be protective against the risk of anaemia.

**Conclusions:**

The risk of anaemia was particularly high among adolescent girls in their early age and among those living in food insecure households. The prevalence of anaemia among adolescent girls is a moderate public health problem. According to the WHO set criteria, the districts could be candidates for intermittent iron and Folic acid supplementation program.

## Background

The World Health Organization (WHO) defines adolescence as the period from 10 to 19 years of age [1]. Nutritional anaemia is the common nutritional problem among adolescent girls in most of the developing countries and its prevalence in low and middle income countries ranges from 13.4 to 62.9% [2, 3]. Ethiopian adolescent girls are not exceptions to this problem and for example, according to the Ethiopia Demographic and Health Survey (EDHS) 2011, 13.4% of women aged 15 to 19 years were anaemic [4, 5]. However, the burden of anaemia among adolescent girls has progressively declined in Ethiopia and the country has made significant improvement in reduction of nutritional anaemia. For example, the prevalence of anaemia among adolescent girls had decreased by 45% % from 24.8% in 2005 [6] to 13.4 in 2011 [6]. However, a study conducted in North East Ethiopia has shown prevalence of anaemia among adolescent girls as high as 22.8% [7].

Adolescent girls are at risk of iron deficiency and anaemia due to various factors including high requirements for iron, poor dietary intake of iron, high rates of infection and worm infestation, as well as pregnancy [8]. Female adolescents in particular are at a higher risk for anaemia compared to their male counterparts [9, 10]. As mentioned above, anaemia among adolescent girls is multi-factorial and the most notable factor is the one that is related to heavy menses. For instances, there are studies that documented girls who started menarche with excessive menstrual bleeding are more likely to develop anaemia [9, 11]. Another important risk factor for anaemia, which was consistently significant in many studies, is low maternal educational attainment [9, 12, 13]. Adolescent girls with lower educational attainment also had higher risk of anaemia [14]. Furthermore, there is evidence that paternal educational status as well predicts anaemia among adolescent girls [15]. These findings indicated that education in general is a very important determinant of anaemia among adolescent girls. Another published risk factor for anaemia among adolescent girls is household socio economic status [7]; this finding is supported by studies conducted across different countries [9, 11, 13].

Inadequate dietary intake of iron-rich food independently determines anaemia among adolescent girls [15, 16]. Adolescents who do not consume eggs, vegetables and meat were found to be at higher risk of anaemia [7]. This could be explained by reduced access to heme iron, which is heavily found in meat and highly bio-available [17]. Infections such as malaria parasitemia and worm infestation are also important contributing factors for anaemia [15]. Malaria related anaemia results from increased destruction of infected and uninfected red blood cells (RBC) as well as impaired erythropoiesis [18].

In nutshell, we have noted variation among studies on the reported magnitude as well as on the relative importance of factors associated with anaemia among adolescent girls. Of the studies that have investigated anaemia among adolescent girls, few of them provided estimates of the magnitude of anaemia for the entire adolescent period (10–19 years) and many of these studies are limited to school going adolescent girls which may potentially underestimate the magnitude of anaemia among adolescent girls. Failure to consider younger and out of school adolescent girls can also result in designing improper intervention modalities. Moreover, adolescent girls requires higher iron intake due to their rapid growth and menstrual losses [19], therefore, the risk of anaemia increases during this period of rapid growth. The consequence of anaemia on maternal and infant health also brings adolescent girls to the spotlight, since adolescent years is one of the windows of opportunities to break the intergenerational cycle of malnutrition.

The WHO Ethiopia country office in collaboration with the government of Ethiopia launched the Accelerating Nutrition Improvement (ANI) project aiming at reducing iron-deficiency anaemia among adolescent girls in 10 selected districts in Ethiopia. This baseline survey was planned to establish the magnitude of anaemia among adolescent girls aged 10–19 years and identify possible delivery channels to provide anaemia control and prevention services to adolescent girls in the target districts. In the present work, we attempted to determine the burden of anaemia among adolescent girls. More specifically, we aimed to: (i) estimate the magnitude of anaemia among adolescent girls in the early, middle and late adolescence period (ii) evaluate adolescent girls’ perceptions on anaemia and preferred delivery channels for Iron and Folic Acid Supplementation, (iii) evaluate and recommend alternative platform for iron supplementation among adolescent girls. The findings from this study will provide insights about the burden of anaemia among the adolescent period and further help design appropriate implementation modalities for iron folic and acid supplementation among adolescent girls.

## Methods

### Study design and period

We employed a community based cross-sectional study design. The study was representative of adolescent girls aged 10–19 years. The study was conducted between October and December 2015.

### Study setting

The WHO country office is supporting the Accelerating Nutrition Improvement (ANI) project. The WHO ANI activities aimed, among others, to improve dietary habits and reducing iron deficiency anaemia among adolescent girls. The project was implemented in ten districts in the regions of Amhara, Oromia, and Southern Nations, Nationalities, and.

Peoples’ Region (SNNPR), in partnership with John Snow, Incorporated and under the overall coordination of the Federal Ministry of Health (FMoH) of Ethiopia. The ten implementation districts were *Beyeda, West Belesa, Laygaynt, Wondogent, Gedebasasa, Derra, Debrelibanos, Angacha, Damotegale, and Boloso bombie*. The baseline study was conducted in three districts (namely *Debrelibanos, Damotegale and Laygaynt)* of the ten WHO Ethiopia country office supported districts.

### Sample size determination

In this baseline study, sample size determination considered any form of anaemia (13.4% of adolescent girls aged 10–19 years are anaemic; ([5], a 95% confidence level, 4% precision, design effect of 1.5, and contingency for non-response at 5%. This gave a sample size of 440 adolescent girls aged 10–19 years per district. Thus, a total of 1320 adolescent girls age 10–19 years in the 3 selected districts were included.

### Sampling procedures

The 3 study districts (*Debrelibanos, Damotegale and Laygaynt* districts) were selected randomly among the 10 WHO ANI project districts. From each district, we randomly selected 6 rural and 1 urban *kebeles* (smallest administrative units in Ethiopia). The selection of *kebeles* for the survey ensured that the sampled households had a balanced geographical spread (lowlands, highlands) within the larger category of rural and urban. The total sample size in each district (*n* = 440) was allocated for the selected *kebeles* proportionally, based on the number of household in the *kebeles*.

In the selected *kebele*, a central point was identified. Data collectors worked in teams of two. Each team of two started out in different directions (the direction decided in a way that can transect the villages within a *kebele*).

The first household was randomly selected from among the first 3 households. From then onwards, data collection teams visited every fourth household. If the selected household did not contain an adolescent girl age 10–19 years, the data collection team moved to the next household (the direction is decided a priori) and resumed their sampling once they identified an eligible household. In households in which there was more than one eligible adolescent girl, the team randomly chose one girl to interview.

### Data collection and instrument

A questionnaire, adapted from the Ethiopian Demographic and Health Survey (EDHS) and relevant literature was developed. The questionnaire was translated into local languages (*Amharic* and *afan oromo* languages). The questionnaire included socio demographic characteristics of the adolescent girls and their respective parents such as education, schooling status, religion, marital status, parental occupation, and family size. Data on household characteristics such as ownership and size of land, type of house and construction materials; availability of fixed assets such as radio, television, phone, bed, and chair and other household items; possession of domestic animals; and access to utilities and infrastructure (sanitation facility and source of water) were collected.

Household food security was measured using the household food insecurity access scale (HFIAS) developed by Food and Nutrition Technical Assistance (FANTA) Project through the Academy for Educational Development [20]. The geographic locations and elevations of visited households were determined using a hand-held global positioning system (GPS) device (Garmin GPSMAP®).

Due to the lack of vital registration systems, we developed a local events calendar to estimate adolescent girls’ year of birth. All enumerators were required to apply the local events calendar to estimate adolescent girls’ age.

The interviews were conducted during weekends i.e. on Saturdays and Sundays in order to capture both in school and out of school adolescent girls in the sample. Adults, preferably mothers or heads of household as appropriate, were interviewed about general household demographic and economic characteristics.

Following the interviews, experienced nurses and lab personnel tested adolescent girls for anaemia using HemoCue B-Haemoglobin analyser. The HemoCue B-Haemoglobin analyser is a portable, rapid and accurate method of measuring haemoglobin. Results are displayed after 45 to 60 s in g/dl on an LCD display. Bio-safety measures such as use of sterile gloves; alcohol/clean water during collection of specimen as well as safe disposal system were employed (used gloves and other materials was collected using safety boxes).

The enumerators and supervisors were trained for three days on general techniques of interviewing and supervision, administration of each item in the questionnaire, hemoglobin measurements, and instruction on ethical treatment of participants. In addition, the questionnaire was pretested in a village, not selected for the study, before the final study began to assess the performance of the study tools. Some revisions were made on the study instruments based on the feedback obtained from the pretest. Interviews were administered by 20 enumerators (experienced nurses and lab personnel) and supervised by three supervisors. The enumerators had a minimum of diploma education (experience in data collection preferable), fluently spoke the local languages, and were residents in the local area or vicinity. The supervisors had a minimum of a bachelor education and previous experience in supervising community based data collection. The supervisors addressed questions and queries of interviewers and corresponded with the investigators whenever necessary. In addition, 2–3 local residents were recruited from each of the districts to guide the data collectors through the villages and ease communication with the villagers. A field guide manual was also developed for use by the interviewers and supervisors.

### Data quality assurance

Three days long training was given for data collectors and supervisors. The focus of the training was on understanding the instrument and interviewing skills, appropriate use of HemoCue for anaemia testing, and GPS operation. Role plays and pretests were done before the actual data collection. The supervisors checked all filled questionnaires for completeness and consistency each day before turning them to the investigator.

The HemoCue instrument is widely used to measure hemoglobin in anaemia surveys. Although the instrument is excellent on its own, data quality is dependent on good blood sample collection (capillary blood sample). The personnel were trained on the correct handling of the instrument and procedures. In order to get accurate and reliable hemoglobin values using HemoCue, standardization exercises were conducted multiple times during training until the performance standard was met. The performance standard is met when the difference in hemoglobin levels between data collectors and expert is less than 0.5 g/dl.

We also checked the quality of the hemoglobin data in the sample by calculating its standard deviation (SD). A smaller SD (1.1–1.5) of hemoglobin is usually considered to denote a better data quality than a larger SD.

Bio-safety measures such as use of sterile gloves and alcohol/water during collection of specimen as well as safe disposal system were employed. Materials like gloves and lancets were collected using safety boxes and were transported for safe disposal, either to be buried or incinerated. For adolescent girls who were anemic, counseling to take Iron and Folic Acid (IFA) supplement and referral to a nearby health facility was arranged.

### Data management and analysis

We used Epi Data Version 3.1 for data entry and Stata 14.0 (Stata Corp, College Station, TX) for cleaning and further analysis.

Descriptive analysis on the general characteristics of the adolescent girls such as age, schooling, marital status, and knowledge on anaemia as well as household characteristics such as household food insecurity, and household dietary diversity was done. In addition, data presentation using tables, graphs and appropriate summary figures were included.

Household wealth: We applied a principal component analysis (PCA) to construct wealth index. In order to construct a relative household’s wealth index, a suite of several socio economic indicators were collected: land ownership, type of house and building materials, availability of fixed domestic assets (i.e. radio, television, bed, chairs and other household items), ownership of domestic animals, source of drinking and cooking water and availability and type of latrine. A relative socio-economic status was constructed by dividing the resulting score into quintiles that indicate poorest, poor, medium, rich and richest households.

#### Household food insecurity and diversity

The household food insecurity (access) was derived from the HFIAS tool. The frequencies of affirmative responses to the HFIAS questions were used to classify households into one of the four categories of food insecurity i.e. food secured, mild, moderate and severe food insecurity. We generated household-level mean dietary diversity score using the sum of all foods (food groups) eaten in the respective household during the day and night prior to the date of the survey. We classified households into three levels (lowest, medium and high) of dietary diversity; a household with a lowest dietary diversity score consumed three or less food groups, a household with a medium dietary diversity score consumed four or five food groups, while a household high dietary diversity consumed six or more food groups.

#### Anaemia prevalence

The hemoglobin (Hb) level was adjusted for high altitude and smoking status before defining anaemia. The adjustment was done to account for a reduction in oxygen saturation of blood. We used the following formula for adjustment of hemoglobin for high altitude.

$$ \mathrm{Hb}\kern0.5em \mathrm{adjustment}\kern0.5em =\kern0.5em \hbox{-} 0.032\ast \left(\mathrm{altitude}\kern0.5em +\kern0.5em 0.003280\right)+0.02\ast +{\left(\mathrm{altitude}\kern0.5em +\kern0.5em 0.003280\right)}^2 $$

where the Hb adjustment is the amount subtracted from each individual’s observed hemoglobin level.

Moreover, hemoglobin adjustments for smoking were done by subtracting 0.3 from individual’s observed hemoglobin level.

Adolescent girls who had an Hb values below 12 g/dL were considered as anemic. Adolescent girls with Hemoglobin values of 11–11.9 g/dL, 8–10.9 g/dL, and < 8 g/dL were categorized as having mild, moderate, and severe anaemia, respectively.

A complex survey data analysis was employed to calculate the district level anaemia prevalence, designating the survey’s primary sampling unit (villages) and strata (urban and rural). The variance was adjusted using Taylor linearized variance estimation method.

Anaemia prevalence was also calculated for each of the districts; among the age groups such as early, middle and late adolescence period; urban and rural residence; and within the following categories: household wealth, food insecurity levels, dietary diversity status, schooling status, agro ecology, heard the term anaemia, and IFA and wealth status, as appropriate.

#### Analysis of the determinants of Anaemia

We run a multivariate logistic regression model using the ‘svy’ command in STATA 14.0 (StataCorp College Station, TX) to ensure that standard errors are adjusted for the complex survey design. This was done to identify factors that could potentially be associated with the occurrence of anaemia among adolescent girls. We selected theoretically relevant variables from the literature for the regression model including household, personal and diet related variable such as household food security, dietary diversity, socio-economic condition, place of residence, adolescent’s age, schooling, smoking and awareness of the term anaemia and IFA tablets.

## Results

We included a total of 1323 adolescent girls aged 10–19 years. Only two adolescent girls refused to give blood for a test due to fear of needle prick.

Table 1 shows a summary of the demographic and other characteristics of adolescent girls in the sample. Majority (65.8%) of the adolescent girls were in the early adolescent period (10–14 years). The mean reported age at interview was 13.6 years. The majority (95.8%) of the adolescent girls were never married, while few of them 3.7% were married, widowed or divorced. Concerning education, 81.5% had some form of formal education in the primary school level (1, 2, 4–9). Although the majority (87.2%) were currently in school, a significant proportion of adolescent girls; (12.6%) were out of school at the time of this survey. Among the out of school adolescent girls, 59.3% had formal education in the primary school level (1, 2, 4–9), 25.5% were Illiterate, whereas 74.9% were single and 20.1% were married (not shown).

**Table 1:** Sample characteristics of adolescents age 10–19 years in the in three districts, Ethiopia 2015 (*N* = 1323)

Characteristics	Number of adolescents(n)	Percent
Reported age (years)		
10–14	871	65.9
15–17	328	24.8
18–19	122	9.2
Current marital status		
Single	1268	95.8
Others (married, divorced, widowed)	49	3.7
Respondent’s education		
Primary education	1082	81.8
Secondary education	178	13.5
College	11	0.8
No schooling	43	3.3
Schooling status		
In school	1154	87.2
Out of school	167	12.6
Residency		
Rural	1101	83.2
urban	222	16.8

Table 2 shows the pattern of food groups consumed in the study sample 24 h prior to the survey. The mean dietary diversity score for the sample is 4.8 (SD = 2.1). Most of the adolescent girls had a lowest or middle diversity scores. About 47% had lowest dietary diversity (consumed three or less food groups) and 42% had a medium dietary diversity score (consumed four or five food groups). Only 11.3% had the highest dietary diversity score. A close look into the consumption of individual food groups indicates that the consumption of animal source food groups that are important sources of iron is very poor. The consumption of milk and milk products, meat, egg and fish was very low (22.5, 5.1, 3.9, and 3.4%respectively). On the other hand, the consumption of cereals, vegetables, oils, grains and roots was relatively higher (91.3, 87.2, 73.1, 67.0 and 61.8%respectively).

**Table 2:** Affirmative responses to food groups consumed in a household 24 h prior to the survey in three selected districts, Ethiopia, 2015

Food groups	n	%(Yes)
bread, rice, noodles, or other foods made from grains	1208	91.3
Any dark green leafy vegetables	452	87.2
Any oil, fats, or butter, or foods made with any of these	967	73.1
Any foods made from beans, peas, lentils, nuts, or seeds	886	67.0
White potatoes, white yams, manioc, cassava, or any other foods made from roots	818	61.8
Cheese, yogurt, or other milk products	297	22.5
fruits or vegetables	155	11.7
meat, such as beef, pork, lamb, goat, chicken, or duck	67	5.1
Eggs	51	3.9
Fresh or dried fish, shellfish, or seafood	32	3.4

### Socio demographic distribution of anaemia

The point and interval estimates of the percentage of adolescents age 10–19 with anaemia, by selected background characteristics is depicted in Table 3. The overall prevalence of any anaemia among adolescent girls in the three districts was 29.2% [95% CI: 24.4, 34.5]. Twenty-five percent of adolescents had mild anaemia, with 3.8% had moderate anaemia, and 0.3% had severe anaemia.

**Table 3:** Percentage of adolescents age 10–19 with anaemia, by area of residence, agro ecology and selected background characteristics, Ethiopia 2015

Background Characteristics	Anaemia status by hemoglobin level			
	Any	Mild	Moderate	Severe
	< 12.0 g/dl	11.0–11.9 g/dl	8.0–10.9 g/dl	< 8.0 g/dl
District				
Debrelibanos	38.3	30.6	7.3	0.5
Damotegale	24.0	22.2	1.6	0.2
Laygaynt	25.2	22.4	2.5	0.2
Place of residence				
Rural	31.6	27.2	4.0	0.2
Urban	19.0]	15.8	2.8	0.4
Reported age (years)				
10–14	31.3	27.1	4.0	0.1
15–17	28.1	24.1	3.7	0.3
18–19	17.2	13.1	2.5	1.6
Schooling				
In school	29.2	25.6	3.3	0.3
Out of school	28.7	21.0	7.2	0.6
Smoking status				
Smokes cigarettes	21.7	17.4	4.3	–
Does not smoke	29.3	25.2	3.8	0.3
Household food insecurity				
Food secured	26.8	23.4	3.2	0.3
Mildly food insecured	28.5	22.6	5.1	0.7
Moderately food insecured	34.4	30.3	3.9	0.3
Severely food insecured	27.6	23.7	3.9	–
Dietary Diversity				
Low	31.3	27.1	3.9	0.3
Medium	28.1	23.4	4.3	0.4
High	23.9	23.3	1.3	–
Agro ecology				
Highlands	31.6	26.6	4.7	0.3
midlands	21.8	21.8	–	–
lowlands	23.9	20.1	3.0	0.7
Wealth quintile				
Lowest (poorest)	32.5	28.0	4.1	0.4
Second (poor)	31.9	28.1	3.8	–
Middle	30.8	26.3	4.5	–
Forth (Rich)	29.0	24.8	3.4	0.8
Highest (Richest)	21.6	18.1	3.0	0.4
Total	29.2[24.4,34.5]	25.2[21.5,29.2]	3.8[2.5,5.7]	0.3[0.1,0.8]

A higher proportion of early adolescent girls, age 10–14 years (31.3%) and middle adolescence, age 15–17 years (28.1%) were anaemic than girls who are in their late adolescence period, age 18–19 years (17.2%).

We documented anaemia prevalence varied by area and residency. A higher proportion of adolescent girls in *Debrelibanos* district were anaemic (38.3, 95% CI: 26.7, 51.4]. The magnitude of anaemia in *Damotegale* and *Laygaynt* was 24.0 and 25.2%, respectively. The prevalence of anaemia among adolescencts also varied by urban and rural residence; a higher proportion of girls in rural areas were anaemic (31.6%) compared to those in urban areas (19%). Also, adolescent girls residing in the highlands had a relatively higher prevalence of anaemia (31.6%) than those girls residing in the lowlands (23.9%).

### Adolescents’ perceptions on anaemia and preferred delivery channels

**Fig. 1:**
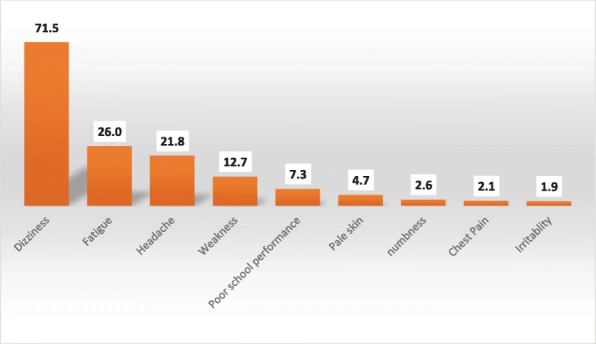
Percentage of Adolescents who knows symptoms of anaemia, Ethiopia 2015

We asked adolescent girls if they have ever heard or know the term anaemia in their local languages. We found that four out of ten adolescent girls have heard about the term anaemia in their local language. We further asked those adolescent girls who heard about the term anaemia if they knew about any symptoms related to anaemia. Figure 1 shows pattern of affirmative responses to selected symptoms of anaemia. Symptoms of anaemia such as dizziness, fatigue, and headache were commonly known by adolescents. Nearly 72% of adolescents knew dizziness, 26% knew fatigue and about 22% knew headache as symptoms of anaemia. However, less than 10% of adolescent girls understood that poor school performance could be a consequence of anaemia.

In addition to this, out of 576 adolescents who heard/knew about anaemia, 35.1% believed that deficiency of iron in foods is a major cause of anaemia. Following, menstrual bleeding (18.9%) and excessive loss of blood (17.4%) were reported as the next main causes of anaemia. Furthermore, we asked adolescents’ whether they knew about IFA supplement, their sources of information, supply and experiences about using them. We found that majority of adolescents did not know about (heard about) IFA. Only 11.2% of adolescent girls had heard about the IFA supplements. Health extension workers and health care providers such as nurses and health officers were primarily sources of information on IFA supplements. Out of the 153 adolescent girls who heard about IFA, only 20.3% had taken the IFA supplement. Most of the adolescent girls who took IFA got the supplements from heath posts (20 out of 31) or from health centers (9 out of 31). We asked these adolescent girls the reason they took IFA; most of them (26 out of 31) reported that they took the IFA because they believed that they were helpful to fill up blood.

We evaluated adolescent girls’ willingness to use IFA and their preferred IFA delivery channel(s). The majority of them; (86.9%) reported that they will definitely be taking IFA to improve physical performance as well as learning and work capacity. Among those who are willing to take IFA, the channels that were most commonly chosen in order of preference were: (1) the health center (43.2%), (2) the health post (35.5%), (3) school clubs (12.0%), (4) at home (8.5%), (5) girls club (3.9%), and (6) the youth centers (1.9%).

### Risk factor analysis for adolescent girls anaemia

Table 4 shows association of adolescents’ and selected characteristics of households with adolescent girl’s Anaemia. The multivariate model indicated that adolescent’s age, place of residence, agro ecology, household food insecurity and knowing (heard) the term “anaemia” were significantly associated with anaemia. We found that the risk of anaemia was significantly high for adolescent girls who lived in *Debrelibanos* (a highland) district. The odds of anaemia among adolescents who lived in *Debrelibanos* district were nearly 2 times than the odds among those who lived in *Damotegale* (AOR 1.95; 95% CI; 1.27–3.01]. Household food insecurity was associated with increased risk of anaemia. We found that adolescent girls who lived in moderately food insecure households were more likely to be anemic than those living in food secured households. (AOR 1.48; 95% CI; 1.05–2.09). The risk of anaemia also varied by the different age brackets during adolescence period. Girls in their early adolescence were more likely to be anemic as compared to adolescent in their late adolescence (OR; 1.98; 95% CI; 1.03,3.82].

**Table 4:** Association of adolescents and selected characteristics of households with adolescent girl’s Anaemia in three districts, Ethiopia 2015

Background Characteristics	Anaemia		Crude OR (95% CI)	Adjusted OR (95% CI)
	Yes (n,%)	No (n,%)		
District				
Damotegale	106 (24.0)	335(76.0)	1.00	1.00
Debrelibanos	169 (38.3)	272(61.7)	1.96[1.19,3.22]	1.95[1.27,3.01]*
Laygaynt	111(25.2)	330(74.8)	1.06[0.66,1.72]	1.02[0.69,1.50]
Place of residence				
Rural	338(31.60	732(68.4)	1.97[1.02,3.81]	1.57[0.84,2.92]
Urban	43(19.0)	205(81.0)	1.00	1.00
Reported age (years)				
10–14	272(31.3)	599(68.7)	2.18[1.18,4.02]	1.98[1.03,3.82]*
15–17	32(28.1)	236(71.9)	1.87[0.96,3.62]	1.95[0.98,3.87]
18–19	21(17.2)	101(82.8)	1.0	1.0
Schooling				
In school	337(29.2)	817(70.8)	1.02[0.64,1.63]	0.93[0.56,1.55]
Out of school	48(28.7)	119(71.3)	1.00	1.00
Smoking status				
Smokes cigarettes	5(21.7)	13(78.3)	1.00	1.00
Does not smoke	331(29.3)	919(70.7)	1.43[0.61,1.26]	0.83[0.22,3.08]
Household food insecurity				
Food secured	200(26.8)	545(73.2)	1.00	1.00
Mildly food insecured	39(28.5)	98(71.5)	1.08[0.72,1.63]	1.08[0.69,1.67]
Moderately food insecured	116(34.4)	221(65.6)	1.43[1.01,2.02]	1.48[1.05,2.09] *
Severely food insecured	21(27.6)	55(72.4)	1.04[0.43,2.52]	0.84[0.38,1.85]
Dietary Diversity				
Low	193(31.2)	424(68.7)	1.39[1.03,1.86]	1.07[0.69,1.65]
Medium	156(28.1)	400(71.9)	1.19[0.75,1.90]	1.09[0.65,1.82]
High	37(24.7)	113(75.3)	1.00	1.00
Agro ecology *				
Highlands	303(31.6)	667(68.4)	1.47[0.78,2.76]	1.30[0.86,1.98]
midlands	45(21.8)	161(78.2)	0.89[0.58,1.36]	0.77[0.63,0.94] *
Lowlands	32(23.9)	102(76.1)	1.00	1.00
Wealth quintile				
Lowest (poorest)	87(32.5)	181(67.5)	1.74[1.01,3.01]	1.39[0.68,1.84]]
Second (poor)	34(31.9)	179(65.1)	1.70[0.92,3.13]	1.26[0.67,2.37]
Middle	82(30.8)	184(69.2)	1.61[0.91,2.85]	1.33[0.60,2.94]
Forth (Rich)	76(29.0)	186(71.0)	1.49[0.93,2.37]	1.91[0.69,2.04]
Highest (Richest)	57(21.6)	207(78.4)	1.00	1.00
Heard the term anaemia				
Yes	136(23.6)	440(76.4)	1.00	1.00
No	250(33.5)	437(66.5)	1.63[1.23,2.15]	1.58[1.09,2.29*]
Heard about IFA tablets				
Yes	43(28.1)	110(71.9)	1.00	1.00
No	341(29.3)	823(70.7)	1.06[0.70,1.89]	0.67[0.43,1.04]

*p < 0.05

We evaluated if knowing anaemia and its meaning had a protective effect from the risk of anaemia. The result showed that risk of anaemia was lower among those adolescents who heard the term anaemia. The odds of anaemia was 60% higher among adolescents who had not of heard the term compared to those who had heard the term anaemia (OR 1.58; 95%CI; 1.09–2.29]. We did not find statistically significant association between the risk of anaemia and characteristics such as household wealth, dietary diversity, schooling status, smoking status and, place (location) of residency.

## Discussion

In this study, we aimed to find out the magnitude and predictors of anaemia among adolescent girls, evaluate adolescent girls’ perceptions on anaemia and preferred delivery channels for Iron and Folic Acid Supplementation and recommend platform for iron supplementation among adolescent girls. The results of the survey indicated that anaemia rates ranged from 24 to 38%, with an average rate of 29%. Adolescent girls in their early adolescence period and those who lived in food insecure households had higher burden of anaemia. Less than half of the girls heard the term anaemia, and about one third knew of the relationship between anaemia and the intake of iron rich foods. The great majority of girls interviewed were willing to take iron-folic acid supplements to improve their health as well as their capacity to learn and to work. Most indicated they would prefer receiving supplements through the health system.

Currently, the magnitude of anaemia among adolescent girls in the studied districts was alarmingly high. We calculated the sample size for this study based on a 13% expected prevalence of anaemia derived from an estimate made from the EDHS 2011. However, our result is more than double of what the EDHS reported in 2011 and from other studies [20, 21]. This variation might be due to differences in the study populations since we have included a wider age range in this study. We also believe that the inclusion of both in school and out of school adolescents unlike other studies might have produced different estimates compared to other studies.

A higher prevalence of anaemia in the early adolescent girl may be attributed to higher prevalence of puberty menorrhagia at the time around menarche. A similar higher prevalence of anaemia in the early adolescence period (10–14 years) than the late adolescence period (15–19 years) was reported in India [21, 22]. Contrary to our finding, a study conducted in India has as well indicated that age and menarcheal status did not affect the prevalence of anaemia among the adolescent girls [23].

We used the dietary diversity score derived from the sum of food groups eaten by household members as proxy indicator for dietary intake and quality. The dietary score for the sampled adolescent girls was very low and the anaemia prevalence varied across these scores. Interestingly, the prevalence of anaemia was still considerably high for adolescents living in households with higher dietary diversity scores. This may be due to the fact that the consumption of iron rich food was generally low among the adolescent girls.

Similar to a study conducted in Bangladesh [24], our findings showed that adolescent from food insure households are likely to suffer from anaemia compared to their food insecure counterparts. Food insecurity can predispose individuals to anaemia through inadequate consumption of micronutrients [25] and the food insure households tend to consume less micronutrient as a result of under consumption of diet or over consumption of energy dense diet that contain less micronutrients which facilitate the bioavailability of iron [26].

The current study also revealed that the prevalence of any form of anaemia was higher (31.6%) in rural adolescent girls as compared to their counterpart in urban area (19%). This finding is analogous to studies conducted in India. The higher prevalence of anaemia among rural adolescent is due to higher possibility of food shortage and thus consumption of food poor in iron and other micronutrients. As anticipated, knowing (heard) the term anaemia was also found to be significantly associated with anaemia status among the adolescent girls. Therefore, nutrition education and counseling can be used to improve the nutrition knowledge of adolescent girls and make them realize healthy diet for healthy living.

According to the WHO classification of public health significance of anaemia in populations on the basis of prevalence estimated from blood levels of hemoglobin, all of the study districts (*Debrelibanos, Damotegale and Laygaynt*) can be classified under the category of moderate public health significance (prevalence of anaemia between 20.0–39.9%). According to the WHO set criteria for IFA supplementation for adolescents, the districts could be candidates for preventive iron supplementation program [27]. We aimed to find out adolescent girls’ perceptions on anaemia and their levels of awareness on perceived causes and symptoms of anaemia. Furthermore, we intended to find out their views and experiences about using and their reported willingness to use IFA. We also considered how IFA supplementation fits within the larger picture of anaemia prevention, and how adolescents will respond to the concept of introducing IFA supplementation as a strategy to combat anaemia. We noted that more than four out of ten adolescents heard the term anaemia. In addition, the girls reported the relationship between anaemia and the intake of iron rich foods, menstrual bleeding and excessive loss of blood. On top of these, adolescents perceived that anaemia is related to poor school performance. These points could be used for messaging when introducing IFA supplementation. In addition, the majority of adolescents do not know about (heard about) IFA but are willing to take IFA. The finding that the great majority of adolescents were willing to take IFA supplements should be taken as an opportunity to initiate a supplementation program. However, choosing delivery channels need to take into consideration the preferences of adolescents. The differing views among adolescents on delivery channels highlight the differences in trust, experiences, privacy and other aspects that adolescents require. It is also worth noting that medicalization of IFA supplements could be one of the possible reasons that adolescents preferred the health system as an outlet for IFA delivery.

The fact that 12% of adolescents were out of school has programmatic implications on choosing the type of delivery channels to use. Programs relying solely on school based delivery channels might miss adolescents who are out of school. Alternative delivery channels including health centers and health posts as well as home to home delivery might be needed to reach of adolescents who are out of school. We recommend a formative assessment with an objective of in depth investigations to explore the appropriateness, feasibility and cost effectiveness of different delivery channels.

## Conclusion

In conclusion, the prevalence of anaemia among adolescent girls was found to be a moderate public health problem. According to the WHO set criteria for IFA supplementation for adolescents, and given the high rate of anaemia found among.

adolescent girls, the districts could be candidates for intermittent iron and Folic acid supplementation program.

## Abbreviations

ANI: Accelerating Nutrition Improvement
EDHS: Ethiopian Demographic and Health Survey
GPS: Global Positioning System
HFIAS: Household food insecurity access scale
IFA: Iron Folic Acid
PCA: Principal Component Analysis
RBC: Red blood Cells
SD: Standard Deviation
WHO: World Health Organization
